# Coexistence of Antineutrophil Cytoplasmic Antibody Associated Vasculitis and Chronic Hepatitis B Virus Infection: A Case Report

**DOI:** 10.7759/cureus.109689

**Published:** 2026-05-26

**Authors:** Krista Maria Bonello, Craig Muscat, Lara Delicata, Angela Borg Cauchi

**Affiliations:** 1 Internal Medicine, Mater Dei Hospital, Msida, MLT; 2 Nephrology, Mater Dei Hospital, Msida, MLT

**Keywords:** anca-associated vasculitis, antineutrophil cytoplasmic antibodies, chronic hepatitis b, hepatitis b virus infection, systemic vasculitis

## Abstract

Antineutrophil cytoplasmic antibody (ANCA)-associated vasculitis (AAV) is a rare autoimmune disease affecting small-to medium-sized vessels. Chronic hepatitis B virus (HBV) infection is typically associated with ANCA-negative vasculitis, making the coexistence of HBV and ANCA-positive vasculitis uncommon and diagnostically challenging.

We report an 84-year-old woman presenting with lethargy and functional decline. Investigations showed elevated inflammatory markers, normocytic anaemia, and haemoproteinuria. Immunology revealed strongly positive myeloperoxidase (MPO)-ANCA, with reactive HBV surface antigen and low viral load (36 IU/mL). Kidney biopsy demonstrated pauci-immune glomerulonephritis with focal fibrocellular crescents.

The patient was treated with oral corticosteroids and entecavir, followed by azathioprine maintenance. Rapid clinical and biochemical improvement was observed, with normalisation of inflammatory markers within one week and resolution of ANCA titres and haemoproteinuria by three months. Eighteen months later, she remains stable with an undetectable HBV viral load.

This case highlights the need to consider AAV in patients with chronic HBV presenting with haemoproteinuria and systemic inflammation. Histological confirmation is crucial due to potential false-positive ANCA results. Combined antiviral and immunosuppressive therapy can be safe and effective with close monitoring.

## Introduction

ANCA-associated vasculitis (AAV) encompasses a group of rare autoimmune diseases characterised by inflammation and damage of small blood vessels, leading to variable organ involvement and clinical manifestations. These conditions are associated with anti-neutrophil cytoplasmic antibodies (ANCA), which are autoantibodies directed against components of neutrophils and are believed to play a central role in disease pathogenesis. The three main ANCA-associated vasculitides are microscopic polyangiitis (MPA), granulomatosis with polyangiitis (GPA), and eosinophilic granulomatosis with polyangiitis (EGPA). [1] MPA is commonly associated with myeloperoxidase ANCA (MPO-ANCA) and is typically described as a pauci-immune vasculitis, meaning there is little or no immune-complex deposition within affected vessels. Although AAV is primarily considered an autoimmune disorder, environmental and external factors, including chronic infections, have been recognised as potential triggers. [2]

Chronic hepatitis B virus (HBV) infection is classically associated with immune-complex-mediated vasculitides, particularly polyarteritis nodosa and cryoglobulinaemic vasculitis, which are usually ANCA-negative. In contrast, the coexistence of chronic HBV infection with ANCA-positive vasculitides such as MPA is rare and may create significant diagnostic uncertainty. [3] This poses important diagnostic and therapeutic challenges, particularly when balancing the need for immunosuppression against the risks associated with chronic viral infection.)

This article was previously presented as an oral presentation at The XI Malta Medical School Conference on December 4, 2025.

## Case presentation

An 84-year-old Caucasian woman with a background of atrial fibrillation, hypertension, and Paget’s disease presented to the emergency department (ED) with a one-month history of progressive lethargy and reduced functional capacity. Her past medical history was notable for a hemicolectomy for adenocarcinoma, spinal discectomy, and microwave ablation of a right renal oncocytoma.

She reported fatigue, lethargy, and intermittent sneezing, but denied features suggestive of systemic vasculitis or infection, including dyspnoea, haemoptysis, weight loss, fever, rash, hearing loss, myalgia, or arthralgia. Regular medications included valsartan, carvedilol, and rivaroxaban.

Investigations

Initial investigations performed in the community demonstrated normochromic normocytic anaemia with a significant inflammatory response. On presentation, haemoglobin was 11.3 g/dL (12.0-15.5 g/dL), with mean corpuscular volume (MCV) 97.5 fL (79.0-97 fL), platelets 309 × 10⁹/L (132-349/L), and creatinine 85 µmol/L (45-84 µmol/L). Inflammatory markers were markedly elevated, with an erythrocyte sedimentation rate (ESR) of 73 mm/hr (2-35 mm/hr) and C-reactive protein (CRP) of 93.5 mg/L (0-5 mg/L). Liver function tests showed mild hypoalbuminaemia (albumin 29.1 g/L; 33-45 g/L) with otherwise unremarkable transaminases and bilirubin.

Cross-sectional imaging of the abdomen and pelvis revealed normal-appearing kidneys, liver parenchyma, and spleen. Urinalysis, however, demonstrated haemoproteinuria with 75 mg/dL protein, an albumin-creatinine ratio of 150 mg/g (1-20 mg/g), and 250 erythrocytes/µL, indicating active renal involvement. The combination of unexplained haemoproteinuria and systemic inflammation prompted further immunological evaluation.

Serology revealed a strongly positive myeloperoxidase-ANCA (MPO-ANCA) >200 U/mL (0-20 U/mL), with a negative anti-glomerular basement membrane (anti-GBM) antibody. Hepatitis B surface antigen was reactive, with a low viral DNA load of 36 IU/mL, consistent with inactive or minimally replicative infection. The full laboratory profile is summarised in Table 1. Retrospective virology from eight years earlier confirmed chronic hepatitis B infection (HBsAg and anti-HBc positive, HBeAg negative, anti-HBe positive), consistent with an HBeAg-negative chronic phase. Hepatitis C and HIV serologies were both negative. Liver elastography demonstrated meta-analysis of histological data in viral hepatitis (METAVIR) F4 fibrosis, indicating advanced chronic liver disease.

**Table 1:** Laboratory Results Marked inflammatory response at presentation (elevated CRP and ESR) with significant improvement following treatment. Renal function worsened initially (rise in creatinine and decline in eGFR at 2 weeks) before partial recovery at 3 months. Urinalysis showed haematuria and proteinuria at presentation with subsequent improvement. ANCA titre was markedly elevated initially with a substantial decline post-treatment. GFR: glomerular filtration rate, ALT: alanine aminotransferase, ANCA: antineutrophil cytoplasmic antibody, DNA: deoxyribonucleic acid.

Investigation	At Presentation	2 weeks post treatment	3 months post treatment	Reference Range and Units
White Blood Cell Count	8.75	15.44	5.44	4.3-11.4 x10^9^/L
Haemoglobin	11.3	13.6	12.3	12.0-15.5 g/dL
Mean Cell Volume	97.5	92.5	99.4	79.0-97.0 fL
Platelets	309	292	246	132-349 x10^9^/L
Creatinine	85	111	82	45-84 µmol/L
Estimated GFR	59	43	62	mL/min/1.73m^2^
C-Reactive Protein	93.5	2.3	2.8	0-5 mg/L
Erythrocyte Sedimentation Rate	73	16	15	33-37 mm/hr
Bilirubin	14.6	14.2	9.1	0-21 µmol/L
Alkaline phosphatase	119	120	82	35-104 U/L
Gamma Glutamyl Transferase	23	56	27	5-36 U/L
ALT	18	26	30	5-33 U/L
Albumin	29.1	35.5	32.2	33-45 g/L
Urinalysis White Blood cells	25	500	Negative	Negative
Urinalysis Proteins	75	25	25	<30mg/dL
Urinalysis Erythrocytes	250	50	25	0-5 cells/HPF
ANCA Titre	>200	178.6	16.1	0-20 U/mL
					Hepatitis B DNA Titre	36	<10	<10	IU/mL

**Figure 1:**
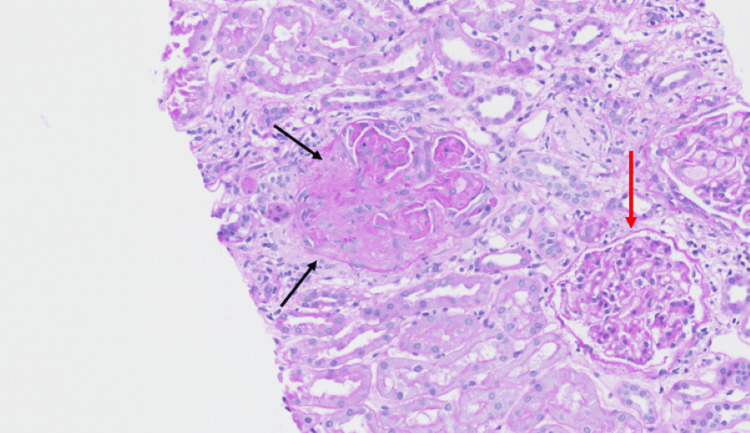
Light microscopy of native kidney biopsy demonstrating focal fibrocellular crescent formation (black arrows) with an adjacent relatively preserved glomerulus (red arrow).

Given the key diagnostic triad of haemoproteinuria, markedly positive MPO-ANCA, and chronic HBV infection, a renal biopsy was performed. Histology, although limited by a suboptimal sample, showed focal fibrocellular crescents with negative immunofluorescence, consistent with pauci-immune crescentic glomerulonephritis in keeping with microscopic polyangiitis. Importantly, no immune complex deposition was identified, supporting ANCA-associated rather than HBV immune-complex-mediated renal disease (Figure 1).

Management

The patient was commenced on oral prednisolone (1mg/kg/day). She was concomitantly started on Entecavir 0.5 mg on alternate days to suppress HBV replication and prevent viral reactivation during anticipated long-term immunosuppression for ANCA vasculitis, and to manage existing liver fibrosis demonstrated on elastography.

Given the absence of a rapidly progressive glomerulonephritis, chronicity of symptoms, and coexistence of chronic HBV in the presence of microscopic polyangiitis, we opted for oral high-dose steroids with a tapering course and an overlap with azathioprine, which was thereafter continued as maintenance therapy together with Entecavir as antiviral prophylaxis.

During her hospitalisation, the patient developed dyspnoea requiring the introduction of low-dose diuretics. A computed tomography pulmonary angiography was performed. This showed the presence of bilateral pleural effusions but excluded pulmonary emboli and pulmonary haemorrhages as other possible differential diagnoses. An echocardiogram and subsequently a cardiac magnetic resonance imaging (MRI) were performed to exclude ANCA-associated cardiac pathology. Whilst the echocardiogram did not elicit any ANCA-related changes, the cardiac MRI reported the presence of mild diffuse myocardial inflammation that may suggest ANCA-driven changes in the context of her biochemical setting.

Follow-up and outcome

**Figure 2:**
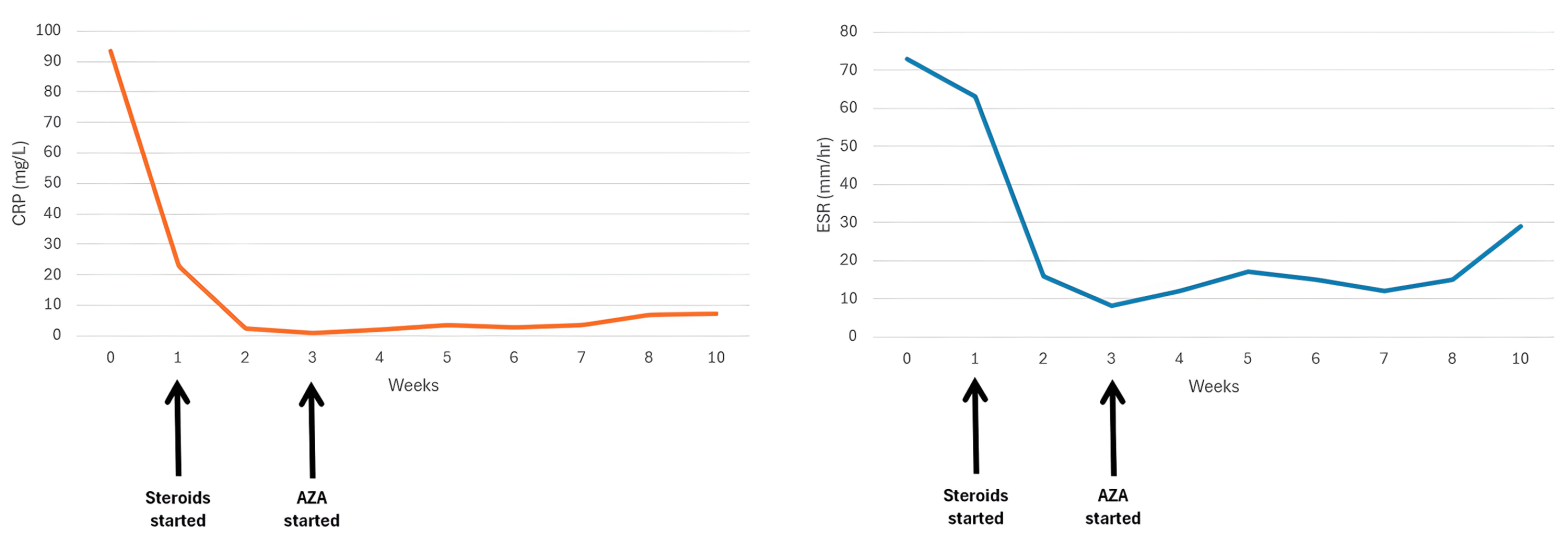
Graphs showing serial CRP and ESR trends. Graphs showing a serial trend in C-reactive protein (CRP) and erythrocyte sedimentation rate (ESR) demonstrating marked inflammatory activity at presentation a rapid decline following initiation of corticosteroids, and sustained suppression after addition of azathioprine at week 3, consistent with disease suppression.

**Figure 3:**
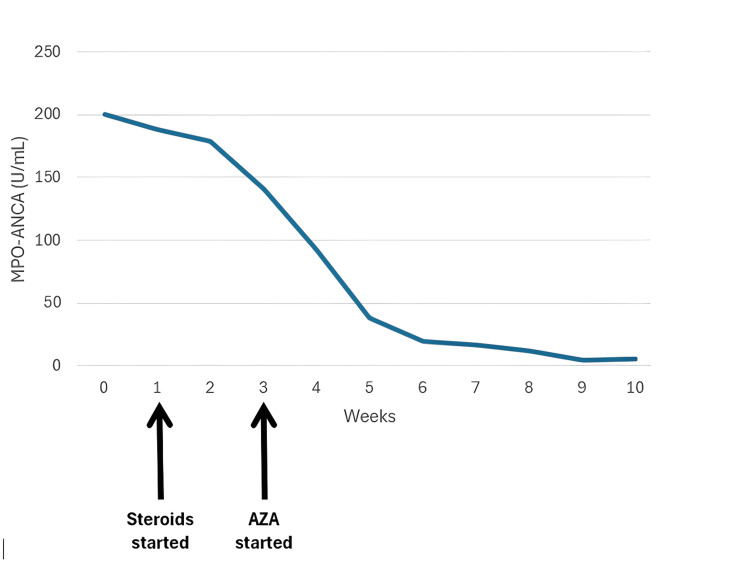
Graph showing serial MPO-ANCA titres. Serial myeloperoxidase (MPO)-ANCA titres demonstrating markedly elevated levels at presentation with a progressive decline following initiation of corticosteroids and further reduction after addition of azathioprine.

One week after corticosteroid initiation, inflammatory markers normalised. Serial monitoring demonstrated persistently low ESR and CRP following corticosteroid and azathioprine therapy (Figure 2). The patient responded well clinically, regaining her baseline level of independence. Within three months of treatment, MPO-ANCA titres normalised and subsequently became undetectable (Figure 3), while haemoproteinuria largely resolved, leaving only trace blood and protein on urinalysis. Follow-up monitoring confirmed persistently undetectable ANCA levels and minimal urinary abnormalities.

**Figure 4:**
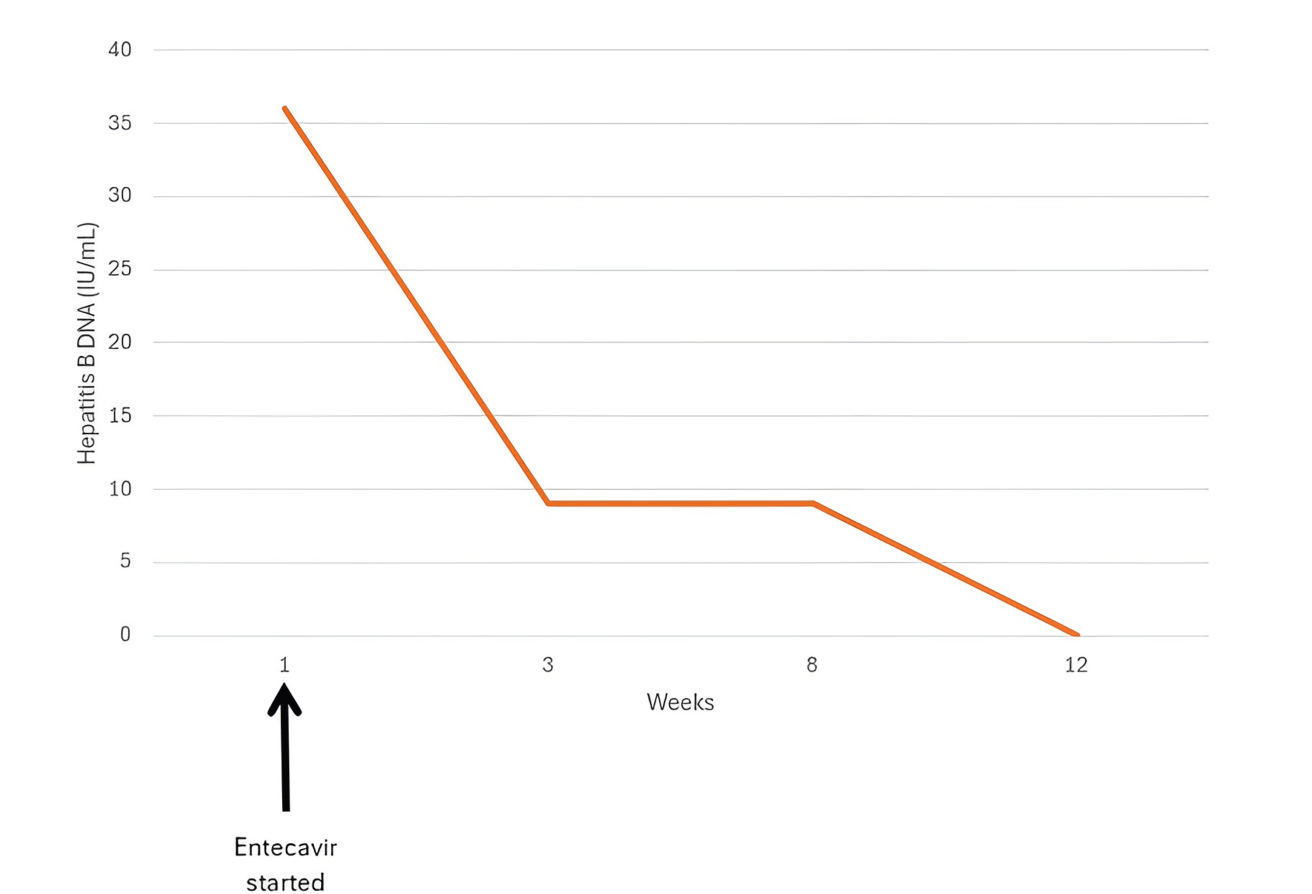
Graph showing serial HBV DNA levels. Serial hepatitis B virus (HBV) DNA levels demonstrating a reduction in viral load following initiation of entecavir, with progressive decline to undetectable levels, consistent with effective antiviral therapy.

Additionally, HBV DNA levels showed a time-dependent decline and became undetectable following initiation of entecavir therapy (Figure 4). At eighteen months follow-up, the patient remained clinically stable on azathioprine and antiviral therapy, with no evidence of disease relapse.

## Discussion

This case describes a rare presentation of MPO-ANCA-positive microscopic polyangiitis (MPA) occurring in a patient with chronic hepatitis B virus (HBV) infection. While polyarteritis nodosa (PAN) remains the vasculitis classically associated with HBV, ANCA-associated vasculitis (AAV) has been infrequently reported in this setting. [1]

Chronic HBV infection is associated with prolonged antigenaemia and immune dysregulation [2], which may lead to non-specific autoantibody formation, including ANCA. Although studies have shown increased detection of MPO-ANCA and PR3-ANCA in HBV-infected populations [3], such seropositivity may be incidental and does not equate to active vasculitic disease. This is clinically important, as isolated ANCA positivity can reflect immune activation rather than pathogenic vasculitis, and may lead to overdiagnosis and inappropriate immunosuppression if not carefully interpreted. In the present case, the initial marked systemic inflammation(ESR 73 mm/hr, CRP 93.5 mg/L) showed rapid improvement within one week of treatment initiation. This early biochemical response is compatible with prompt corticosteroid therapy in AAV and supports disease responsiveness; however, it also limits the ability of inflammatory markers alone to reliably reflect disease activity over time. This is further emphasised by the decline in MPO-ANCA titres, which, while supportive of treatment response, are recognised to have imperfect correlation with clinical disease activity and should not be interpreted in isolation.

Importantly, the integration of renal findings strengthens the diagnostic interpretation. The presence of haemoproteinuria and rising albumin-creatinine ratio corresponded directly with biopsy evidence of active pauci-immune necrotising glomerulonephritis with crescents, establishing a pathological correlate for the urinary abnormalities. The lack of immune complex deposition further differentiates this process from HBV-associated immune-complex vasculitis, reinforcing AAV as the primary mechanism of renal injury despite concurrent HBV infection.

Cardiac MRI findings suggestive of myocardial inflammation were considered in the broader context of systemic small-vessel involvement, although alternative aetiologies, including age-related or non-vasculitic inflammatory change, remain possible. In this case, multi-organ assessment was therefore interpreted collectively rather than in isolation to support a systemic vasculitic process.

The low HBV DNA level (36 IU/mL) is consistent with inactive or minimally replicative infection and has been reported in other cases of HBV-AAV coexistence [1,4 ], supporting the interpretation of coexistence rather than direct viral causation. Nevertheless, a causal relationship cannot be established, and HBV-related immune activation remains a plausible contributing factor to ANCA positivity.

These findings highlight the importance of integrating serial laboratory trends, histopathology, and imaging rather than interpreting each dataset independently. In particular, biopsy confirmation was pivotal in shifting diagnostic certainty toward true AAV, significantly increasing the likelihood of vasculitis over incidental ANCA seropositivity in chronic HBV infection.

MPA is characterised by pauci-immune necrotising small-vessel inflammation, commonly involving glomeruli, in contrast to PAN, which is immune complex-mediated and affects medium-sized arteries with stronger HBV association. The pathogenic role of ANCA involves neutrophil activation, complement amplification, and endothelial injury, leading to small-vessel damage. [5]

Management of AAV in HBV infection requires balancing immunosuppression against viral reactivation risk. Antiviral prophylaxis is essential, and treatment intensity should be guided by organ involvement and disease severity. In this case, the absence of rapidly progressive renal failure and the patient’s frailty supported a more measured immunosuppressive strategy with close monitoring.

Only a limited number of case reports have described the coexistence of AAV and chronic HBV infection [4]. Ijaz et al. [6] and Meng et al. [7] reported cases involving PAN and MPA in HBV-infected patients, highlighting overlapping clinical phenotypes and multi-organ involvement. Similarly, Joshi et al. [8] described a patient with HBV seropositivity and c-ANCA in the context of acute transverse myelitis, suggesting a possible vasculitic process responsive to combined immunosuppressive and antiviral therapy. More recently, Li et al. [9] reported AAV with glomerulonephritis in a patient with chronic HBV infection. While these reports raise the possibility of an association, they do not establish causality, and coexistence remains a more cautious interpretation.

Additionally, isolated reports such as that by Gil et al. [10] have described vasculitic syndromes, including granulomatosis with polyangiitis (GPA) and PAN, following HBV vaccination. While such observations suggest a potential immunological link, they remain rare and insufficient to infer a direct etiological relationship [11]. Further studies are needed to better define the prevalence, clinical significance, and optimal management of AAV in patients with chronic HBV infection. Improved understanding of this coexistence will help inform evidence-based strategies,

Further studies are needed to better define the prevalence, clinical significance, and optimal management of AAV in patients with chronic HBV infection. Improved understanding of this coexistence will help inform evidence-based strategies, particularly regarding the safe use of immunosuppression in this complex clinical setting.

## Conclusions

This case highlights a rare coexistence of ANCA-associated vasculitis in a patient with chronic hepatitis B virus infection, presenting with non-specific symptoms, systemic inflammation, and active urinary sediment. In patients with chronic HBV, a positive ANCA should prompt thorough evaluation, as seropositivity alone may reflect immune activation rather than true vasculitis. Histological confirmation remains essential to establish the diagnosis and guide management. Clinicians should maintain a high index of suspicion for AAV in patients with haemoproteinuria and inflammatory features, even in the context of chronic infection. A combined approach using antiviral therapy alongside carefully selected immunosuppression was effective in achieving a favourable clinical outcome, consistent with previously reported case experiences, although the inherent limitations of case-based evidence should be acknowledged.

## References

[1] Bhagoowani S, Devi U, Munir A, Hasnain U, Iqbal J (2024). Antineutrophil cytoplasmic antibodies (ANCA)-associated vasculitis in chronic Hepatitis B: unraveling the immune puzzle - a rare case report with review of literature. IDCases.

[2] Konstantinov KN, Ulff-Møller CJ, Tzamaloukas AH (2015). Infections and antineutrophil cytoplasmic antibodies: triggering mechanisms. Autoimmun Rev.

[3] Calhan T, Sahin A, Kahraman R, Altunoz ME, Ozbakır F, Ozdil K, Sokmen HM (2014). Antineutrophil cytoplasmic antibody frequency in chronic hepatitis B patients. Dis Markers.

[4] Li MR, Li LY, Tang J, Sun J (2025). Chronic hepatitis B triggering antineutrophil cytoplasmic antibody-associated vasculitis complicated by glomerulonephritis: a case report. World J Clin Cases.

[5] Massicotte-Azarniouch D, Herrera CA, Jennette JC, Falk RJ, Free ME (2022). Mechanisms of vascular damage in ANCA vasculitis. Semin Immunopathol.

[6] Ijaz SH, Taseem S, Usman K (2018). Relapsing hepatitis B-associated vasculitis with features of polyarteritis nodosa and cANCA-associated vasculitis. JOJ Case Stud.

[7] Joshi U, Subedi R, Gajurel BP (2017). Hepatitis B virus induced cytoplasmic antineutrophil cytoplasmic antibody-mediated vasculitis causing subarachnoid hemorrhage, acute transverse myelitis, and nephropathy: a case report. J Med Case Rep.

[8] Meng Z, Cui W, Arend L, Mikdashi J (2021). Hepatitis B virus infection associated with polyarteritis nodosa and microscopic polyangiitis. BMJ Case Rep.

[9] Qasim A, Patel JB (2026). ANCA-associated vasculitis. http://Availableathttps://pubmed.ncbi.nlm.nih.gov/32119259/(Accessed:11February).

[10] Gil E, Lutalo P, D'Cruz D (2011). Systemic vasculitis: a dual diagnosis?. BMJ Case Rep.

[11] Zhang Q, Shi B, Zeng H (2020). Antineutrophil cytoplasmic antibodies (ANCA)-positive patient with infective endocarditis and chronic hepatitis B virus: a case report and review of the literature. J Med Case Rep.

